# Table Olives More than a Fermented Food

**DOI:** 10.3390/foods9020178

**Published:** 2020-02-12

**Authors:** Giorgia Perpetuini, Roberta Prete, Natalia Garcia-Gonzalez, Mohammad Khairul Alam, Aldo Corsetti

**Affiliations:** Faculty of BioScience and Technology for Food, Agriculture and Environment, University of Teramo, 641000 Teramo, Italy; giorgia.perpetuini@gmail.com (G.P.); rprete@unite.it (R.P.); ngarciagonzalez@unite.it (N.G.-G.); mohammadkhairul.alam@studenti.unite.it (M.K.A.)

**Keywords:** table olives, starter cultures, LAB, yeasts, fermented food, probiotic table olives, non-dairy probiotics

## Abstract

Table olives are one of the oldest vegetable fermented foods in the Mediterranean area. Beside their economic impact, fermented table olives represent also an important healthy food in the Mediterranean diet, because of their high content of bioactive and health-promoting compounds. However, olive fermentation is still craft-based following traditional processes, which can lead to a not fully predictable final product with the risk of spontaneous alterations. Nowadays, food industries have to face consumer demands for safe and healthy products. This review offers an overview about the main technologies used for olive fermentation and the role of lactic acid bacteria and yeasts characterizing this niche during the fermentation. Particular attention is offered to the selection and use of microorganisms as starter cultures to fasten and improve the safety of table olives. The development and implementation of multifunctional starter cultures in order to obtain heath-oriented table olives is also discussed.

## 1. Introduction 

Table olives are defined as “the sound fruit of varieties of the cultivated olive trees (*Olea europaea* L.) that are chosen for their production of olive whose volume, shape, flash-to-stone ratio, fine flesh, taste, firmness, and ease of detachment from the stone make them particularly suitable for processing; treated to remove their bitterness and preserved by natural fermentation; or by heat treatment, with or without the addition of preservatives; packed with or without covering liquid” [1]. Table olives are considered one of the oldest fermented vegetables in the Mediterranean basin and are an important element for the economy of several countries. Their production exceeded 2.9 million tons in the 2017/2018 season and the main producers are Spain, Egypt, Turkey, Algeria, Italy, Greece, and Portugal [2]. However, their production is increasing also in other countries, such as South America, Australia, and the Middle East [2]. Moreover, in 2010 they have been added in the Healthy Eating Pyramid of the Mediterranean diet (https://dietamediterranea.com/), because of their high content of bioactive compounds, dietary fibers, fatty acids, and antioxidants [3].

The olive fruit is a drupe which cannot be consumed directly from the tree because of the presence of a bitter compound called oleuropein. The bitterness can be removed by alkaline treatment, or by brining/salting, fermentation, and acidification [4]. According to the International Olive Oil Council (IOOC) [1], the main goals of olive processing are to improve their sensory characteristics and to ensure safety of consumption. The “trade standard applying to table olives” [1] describes the type of preparation of table olives; however, some traditional processes are still applied, such as the Castelvetrano system. This method is diffused in Sicily and mainly is based on the exploitation of the Nocellara del Belice variety. Only olives of more than 19 mm in diameter are used, which are placed in vessels and treated with a 1.8%–2.5% NaOH solution for one hour. After that, 5–8 kg of salt are added, and the olives are maintained in this brine for 10–15 days. A mild washing step is performed to avoid the total elimination of lye [5].

The main trade preparations are reported in Table 1.

## 2. Table Olives Associated Microbiota

Olive fermentation is a complex process involving a wide array of microorganisms and mainly lactic acid bacteria (LAB) (e.g. *Lactobacillus plantarum* and *Lactobacillus pentosus*) and yeasts (*Saccharomyces cerevisiae*, *Wickerhamomyces anomalus*, *Candida boidinii*, etc.) [6]. Their enzymatic activities shape the characteristics of the final products, e.g., flavor, texture, and safety [6]. Moreover, strains isolated from table olives show specific probiotic traits and are able to adhere to the fruit’s epidermis, which could thus be ingested by consumers, turning olives into a carrier for these beneficial microbes [7].

**Figure 1:**
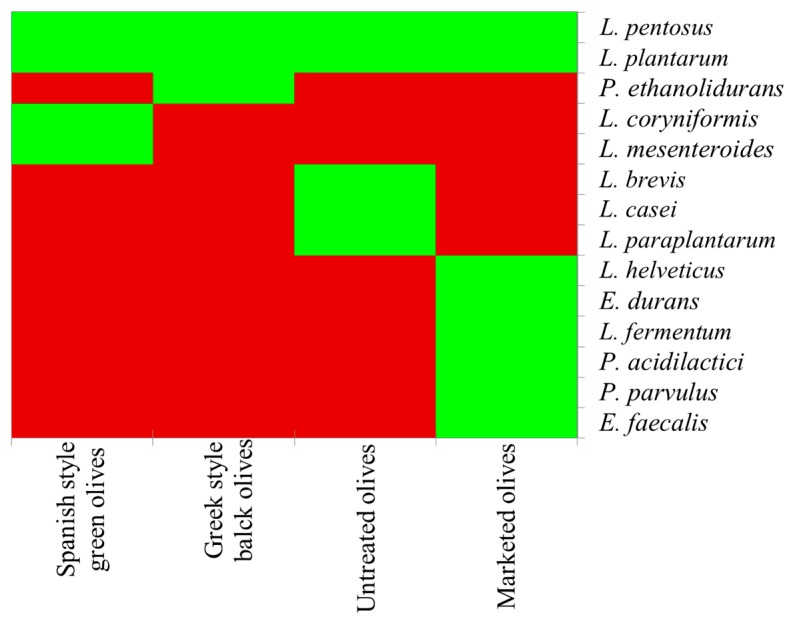
LAB species detected in table olives. The green color indicates the presence of the species, while red its absence. Spanish-style olives are debittered through the addition of lye. In the Greek style, olives are put directly in brine and oleuropein is removed by the enzymatic activities of indigenous microorganisms.

The role of LAB during olive fermentation has been investigated in detail [8,9,10,11,12,13,14,15]. The majority of studies indicated that *L. plantarum* and *L. pentosus* are the main LAB isolated from table olives [10,13,15,16]. They are facultative heterofermentative; therefore, they can produce different end products, such as lactic acid, acetic acid, and carbon dioxide or only produce lactic acid depending on the environmental conditions [13]. Hurtado et al. [13] highlighted that *L. plantarum* produced a higher amount of acetic acid during olive fermentation than *L. pentosus*, suggesting the lower ability of the latter species to preserve a homofermentative metabolism under stress conditions. The main species are reported in Figure 1. LAB are the main bacteria responsible of olive debittering thanks to their enzymatic reservoir (β-glucosidase and esterase). *L. pentosus* is characterized by a strong β-glucosidase activity [11]. This enzyme catalyzes oleuropein degradation and the release of glucose and aglycone. This last compound is converted to non-bitter compounds, such as elenolic acid and hydroxytyrosol, by an esterase [17]. They also play a key role in the decrease of pH and provide microbiological stability to the final product as well as an extended shelf life. The production of lactic acid induces an acidification of brine that prevent the growth of spoilage microorganisms and pathogens [17,18].

Yeasts can play a double role during olive fermentation; in fact, they are associated with the production of volatile compounds (e.g., alcohols, ethyl acetate, and acetaldehyde) and metabolites that improve the taste and aroma and the preservation characteristics of this fermented food. Moreover, they can enhance LAB growth by the release of nutritive compounds, either synthesizing vitamins, amino acids, and purins, or by metabolizing complex carbon sources [19,20,21]. Finally, they show esterase and lipase activities. The first one improves the olive taste since it is involved in the production of esters from free fatty acids, while the second one changes the free fatty acids composition of olives improving the characteristics of the final product [22]. On the other hand, yeasts may cause gas-pocket formation and softening of the olive tissue, or even package bulging, clouding of the brines, and production of off flavors and odors [20]. 

Microbiological studies revealed that *W. anomalus*, *S. cerevisiae*, *Pichia kluyveri*, and *Pichia membranifaciens* are the yeast mainly present in olive brine [6,20,23,24]. *S. cerevisiae* and several species of the *Pichia* genus showed antioxidant activity which protects fruits from oxidation and peroxide formation [21]. Hernandez et al. [21,25] underlined the relevance of *W. anomalus* during olive fermentation. In fact, it presents β-glucosidase activity, as well as produces anti-oxidant compounds and killer toxins against human pathogens and spoilage microorganisms. 

Moreover, *D. hansenii*, *P. membranifaciens*, and *W. anomalus* showed strain-specific killer activity against spoilage yeasts [20,23,25,26].

A recent study started to study the biogeography of the microbial communities associated with Spanish-style green olive fermentations [27]. The authors studied the microbial biodiversity of 30 ten-ton fermenters of three different fermentations yards (*patios*) during the fermentation process. Some species were constant, representing the core microbiota of this area. *L. pentosus, Pediococcus parvulus, Lactobacillus collinoides/paracollinoides, Lactobacillus coryniformis, L. plantarum, Pichia manshurica*, and *Candida thaimueangensis* were found in every *patio.* In particular, cosmopolitan strains belonged to the following species: *L. pentosus*, *P. parvulus*, *L. collinoides/paracollinoides*, and *P. manshurica*.

## 3. Microbial Spoilage of Table Olives

Olive fermentation is still craft-based; therefore, it is not fully predictable, and some alterations can occur. During the first phase of Spanish fermentation, the Gram-negative bacteria prevail. This phase lasts until LAB grow up inducing a decrease in pH. If this reduction is not too fast, “gas pockets”, resulting in the softening and breakage of the cuticle, can appear [28]. A high pH can also favor the development of *Clostridium* spp., which could induce a putrid or butyric fermentation, which cause the appearance of off-flavors and off-odors [28].

The softening of olive drupe is another alteration due to the development of pectinolytic yeasts (e.g., *P. manshurica*, *Pichia kudriavzevii,, Saccharomyces oleaginosus*, etc.), molds (*Aspergillus niger*, *Fusarium* spp., and *Penicillium* spp.) and some bacteria (*Bacillus* spp., *Aerobacter* spp., etc.) [29]. These microorganisms release degrading enzymes, which act on pectic substances and cellulose, hemicellulose, and polysaccharides, causing the loss of the structural integrity of the olive drupe [28,29].

Seville-style table olives can undergo a defect called “white spot”. These spots develop between the skin and the flesh and are associated to the development of some *L. plantarum* strains [30]. 

Finally, when the final product is not pasteurized *Propionibacterium* can develop, producing acetic and propionic acids. This alteration is called “zapateria” and cause an increase in volatile acidity and the formation of cyclohexanecarboxylic acid [31] and the production of biogenic amines, such as cadaverine and tyramine [32].

## 4. Table Olives’ Starter Cultures 

The use of starter cultures for table olives fermentation is highly recommended [17]. An appropriate inoculum reduces the effects of spoilage microorganisms, inhibits the growth of pathogenic microorganisms, and helps to achieve a controlled process, reducing debittering time and improving the sensorial and hygienic quality of the final product [17,33,34,35]. Two different types of starter cultures can be applied. Natural starter cultures are made up of microorganisms that spontaneously colonize the raw materials [3]. Their composition is often not reproducible; however, they guarantee a high biodiversity, which contributes to enrich the final product with particular sensory characteristics mostly linked to the region of origin of the raw material itself [36]. On the other hand, selected starter cultures provide numerous advantages (Table 2). They are usually represented by a single strain or by a mixture of strains previously selected on the basis of specific features: A high survival capacity in the fermentation environment (low pH, high concentrations of salts, and low fermentation substrates); high acidifying activity (through organic acid production); the ability to hydrolyze phenolic compounds (such as oleuropein); as well as the possibility of producing volatile molecules and/or specific enzymatic activities that contribute positively to the development of the sensory profile of the final product [17]. Another important characteristic of a starter culture is its ability to dominate the indigenous microbiota [17]. Dominance of the starter culture would be exerted by its fast and predominant growth under fermentation conditions and/or its ability to produce antagonistic substances [37]. In addition, for commercial purpose, it is necessary that starter cultures resist the freezing or freeze-drying process [17].

Despite these advantages, the application of starter cultures for olive fermentation is still limited [6]. Some of the most important olive varieties are still processed without their addition [3].

Among LAB species, the most often proposed as starter cultures are *L. plantarum* and *L. pentosus* [15,17,38], used alone or in combination with other bacterial or yeast species (Table 3 and Table 4). 

Several studies were conducted to drive the fermentation processes and to improve the quality and sensory profiles of different table olive cultivars using both autochthonous and commercial oleuropeinolytic strains belonging to the *L. plantarum* group [33,34,39,40,41,42,43,44].

Different *L. pentosus* and *L. plantarum* starter cultures have been found to dominate and improve the fermentation process of green table olives in terms of processing time, microbiological quality, color stability, and aroma profile [39,40,41]. 

A strain of *Lb. pentosus* (1MO) was used as a starter to shorten the debittering process of different cultivars (cv. Itrana and Leccino) at the pilot and industrial scale [45]. The use of the selected strain *L. pentosus* (1MO) significantly improved the quality and safety aspects of the fermented table olives, allowing to successfully end the fermentation process within eight days, while more than one week or even months are usually required for biological spontaneous fermentation [46,47,48]. 

Recently, a starter culture made up of two *L. pentosus* strains was successfully used to debitter green table olives (cv. Itrana) [35] and was patented (Patent N0. 0001428559).

Interestingly, the use of *L. plantarum* strains as starter strains has been investigated also for the ability to positively affect the fermentation process in term of quality preservation and stability during storage. Sherhai et al. [42] found a protective effect of *L. plantarum* on fatty acid oxidation and peroxidation processes, as well as a strong antioxidant activity during the Spanish-style fermentation process. In line with that, a recent study on inoculated Nocellara Etnea table olives with six different starter cultures made up of *L. plantarum, L. pentosus*, and *L. paracasei* confirmed the dominance of *L. plantarum* during fermentation and its positive impact on table olives [34]. 

Furthermore, a sequential inoculation strategy has been proposed as a promising biotechnological tool to produce low salt Nocellara Etnea table olives. The authors reported on the use of a β-glucosidase-positive strain, *L. plantarum* strain, followed after 60 days by the inoculum of a *L. paracasei* probiotic strain. This strategy reduced the processing time, and positively affected the polyphenol content and sensory profile of the final product, which was characterized by a low salt concentration (5%) [43]. 

In recent years, several studies focused on the development of yeast starter cultures, both alone and in combination with LAB [20,23,26,49,50,51]. *L. plantarum* and *L. pentosus* strains have been used with excellent results in combination with an autochthonous *Wickerhamomyces anomalus* strain to accelerate the fermentation of Bella di Cerignola table olives [33]. A functional starter strain of *L. pentosus*, with and without *P. membranifaciens*, was successfully used to drive fermentations of Conservolea black olives, which allow producing a functional product with an improved sensory profile [52].

A sequential inoculation strategy (firstly yeasts, then bacteria) was developed by Tufariello et al. [53]. In particular, the authors tested different yeast species (*S. cerevisiae*, *D. hansenii*, and *W. anomalus*) in combination with *L. plantarum* and *Leuconostoc mesenteroides* in order to improve the sensory and organoleptic properties of table olives. Pilot-scale fermentations with the sequential inoculation of LAB and yeast strains reduced the fermentation time (from 180 to 90 days), as well as improved the organoleptic characteristics of the final product [53].

Other yeasts species, such as *Debaryomyces* spp., *Pichia* spp., and *Rhodotorula* spp., were recently investigated in order to select the appropriate strains to use in combination with LAB [3,54] (Table 2). 

Bonatsou et al. [54] selected *P. guilliermondii* and *W. anomalus* among several yeast strains, isolated from black table olives, and screened for their technological and probiotic properties as promising multifunctional starters to use in real olive fermentations. The use of yeasts is also linked to their ability to favor the formation of multispecies biofilms on biotic (drupes) and abiotic (fermenter vats) surfaces [3]. Several studies showed the ability of some yeast species, such as *D. hansenii*, *Geotrichum candidum*, *P. guilliermondii*, and *W. anomalus*, to form biofilm and create a positive environment for *L. pentosus* growth [7,23,55,56,57,58,59].

Recently, the application of autochthonous strains has arisen to face consumers’ demand for more traditional products with a unique sensory profile and peculiar organoleptic properties [60]. Autochthonous strains, being well adapted to the raw material conditions, can easily lead the fermentation process by dominating the table olives microenvironment [3,51]. However, only few studies report the application of autochthonous starter cultures [36,53,61,62]. Martorana et al. [36] used autochthonous starter cultures as a “*Pied de cuve*” to ferment Nocellara del Belice olives [36]. The application of autochthonous starter cultures could be useful for achieving IGP and PDO (Protected Designation of Origin) product specifications, linking the fermented final product to the region where it comes from [3].

## 5. New Trend in Olive Production: Probiotic Table Olives

The concept of functional food was born in Japan around the 1980s; in 1991, the acronym FOSHU (Foods for Specified Health Use) was coined. Nowadays, the accepted definition is the one recognized by the European Union Food Information Council (EUFIC), based on which functional foods are defined as “foods similar in appearance to conventional foods that are consumed as part of a normal diet, and have demonstrated physiological benefits and/or the capacity to reduce the risk of chronic disease beyond their basic nutritional functions” [88]. Probiotics and prebiotics represent the most-used strategies for the production of functional foods [89,90,91,92,93,94]. Probiotics are defined as “live microorganisms which, when administered in adequate amounts, as part of a food or a supplement, confer a health benefit on the host” [95]. Generally, probiotics are bacteria isolated from human sources, mostly from the gastrointestinal tract [96], and mainly belong to *Bifidobacterium* and *Lactobacillus* genera [88]. Indeed, it has been recently showed that also naturally occurring food-associated microbes can reach the gut as viable cells, interact with the human host, and potentially provide benefits to gut health [97]. In this context, a diet may represent not only a source of nutrients to the body, but can be also a vehicle of exogenous microorganisms with positive effects on human health [98,99]. 

Table olives represent a wide reservoir of putative beneficial microbes. Thus, several studies have been conducted to assess the probiotic effects of strains isolated from different fermented olives cultivars and/or already used as starter cultures, belonging to the most widely spread species *L. plantarum* and *L. pentosus*, as well as to species less frequently used, such as *L. paracasei*, *L. casei*, and *L. paraplantarum* [43,85,100,101,102,103,104,105,106]. Some studies revealed that some LAB strains isolated from table olives were able to adhere to porcine jejune epithelial cells IPEC-J2 and produced antimicrobial compounds able to inhibit *Helicobacter pylori*, *Propionibacterium* spp., and *Clostridium perfringens* [10,70,107,108,109]. Probiotic potential, based on the ability to outcompete foodborne pathogens for cell adhesion, was also characterized in several *L. pentosus* isolated from different table olive cultivars (i.e., Nocellara del Belice and Aloreña green table olives) [103,110]. Strains isolated from both cultivars showed the ability to adhere to human intestinal epithelial Caco-2 [110] and vaginal cells [103], as well as the ability to auto-aggregate and co-aggregate with pathogenic bacteria, to ferment some prebiotics, and to in vivo exert protective effects in *Caenorhabditis elegans* [103,110]. Beside antimicrobial activity, different strains of *L. pentosus* and *L. plantarum* isolated from table olives stimulated the release of pro-inflammatory (IL-6) and anti-inflammatory (IL-10) interleukins on macrophages, suppressed the secretion of IL-8, and showed anti-proliferative activity on the HT-29 cell line [111].

Table olives of different cultivars have already been validated as a promising carrier for delivering different probiotics strains into the human GI tract [112] (patent application EP2005/0104138 [9,113]. Table olives can be considered an ideal matrix for the survival of probiotics due to the nutrients released by the fruits and the fact that drupes are coated with a hydrophobic epicuticular wax that promote microbial adhesion [6,7,52,56,112,113,114,115,116].

The probiotic *L. paracasei* strain LMGP22043 was able to colonize the human gut, positively influencing fecal bacteria and biochemical parameters [113]. Lavermicocca et al. [112] used table olives as carrier for the probiotic *L. paracasei* strain IMPC2.1. The strain was recovered in human feces after fermented olive intake, confirming the possibility to use table olives as carrier of probiotics into the human gastrointestinal tract [112]. An autochthonous potential probiotic *L. pentosus* strain [23,75,103] showed to be able to survive for 200 days in packed olives, confirming the possibility to incorporate probiotic strains and thus produce functional table olives [76]. 

The genetic basis of LAB strains adhesion on olive surfaces is still in its infancy. Perpetuini et al. [115] revealed that the sessile state represented the prevailing *L. pentosus* life-style during table olive fermentation and that the three genes *eno*A1, *gpi* and *oba*C were necessary in *L. pentosus* to form an organized biofilm on the olive skin. The first two genes encoded for cytosolic enzymes involved in the glycolysis pathway and in the adhesion to some specific components of olive skin, while *oba*C for a putative fatty acid binding protein of the DegV family, which could bind some lipids of the epicuticular wax. More recently, Pérez Montoro et al. [116] analyzed the adhesion to mucin of *L. pentosus* strains isolated from Aloreña green table olives. They revealed the presence of four moonlighting proteins over-produced in adhesive strains, which were not produced in non-adhesive strains. These proteins were involved in the glycolytic pathway (phosphoglycerate mutase and glucosamine-6-phosphate deaminase), stress response (small heat shock protein), and transcription (transcription elongation factor GreA). A new in silico approach confirmed that moonlighting proteins are involved in the adhesion to both the extracellular matrix (i.e., olive surface) and host cells, as well as in host immunomodulation [117]. Due to the importance of the genetic background on health-promoting traits, Calero-Delgado et al. [118] recently published the draft genome sequences of five *L. pentosus* strains isolated from biofilms on the skin of green table olives. In particular, most of the strains evaluated harbored two copies of the *luxS* gene, involved in the production of the universal bacterial communicator autoinductor-2. Genes encoding for bacteriocin, exopolysaccharide, and MucBP proteins, which could play an important role in microbe-eukaryote cell adhesion, were also found [118]. The main feature of these studied strains was their ability to adhere to the surface of olives during fermentation, forming biofilms, and turning table olives into carriers of beneficial microorganisms to consumers [114,115,119].

Recently, different studies have been focusing on the yeast microbiota associated with table olives fermentations in order to find potential probiotic candidates to be used as starter cultures [23,26,54,100,101,102,103,104,105,106]. *Saccharomyces boulardii* represent the only yeast with claimed probiotic effects [120]. Evidences of other yeast species showing probiotic features, mainly associated with table olive microbiota, such as *D. hansenii, T. delbrueckii, K. lactis, and S. cerevisiae*, are emerging [121,122,123,124].

Different *Torulaspora delbrueckii* and *Debaryomyces hansenii* strains have been found to survive in the presence of high bile salt concentrations and low pH values, as well as to have antimicrobial activity against foodborne pathogens [26]. Furthermore, Silva et al. [125] found some *P. membranifaciens* and *Candida oleophila* strains within a native yeast population of Portuguese olives to be promising candidates as multifunctional starter cultures, by having both technological (oleuropeinolytic activity) and beneficial potential (vitamins production, mycogenic, and antimicrobial activities). 

In this context two important issues to be considered are the assessment of technological factors influencing the survival of probiotic starter cultures and the starter effect on olives’ sensory profile. Rodríguez-Gómez et al. [77] evaluated the effects of inoculation strategies on the survival of *L. pentosus* TOMC-LAB2—a potential probiotic strain when used as a starter culture in large-scale fermentations of green Spanish-style olives. They proposed an inoculation immediately after brining to reduce the presence of initial natural microbiota, the re-inoculation to replace the possible initial died starter and an early processing in the season when starter survival is higher. Concerning the second aspect, a recent study analyzed the organoleptic characteristics of traditional, spontaneously fermented green table olives and green table olives inoculated with *L. pentosus* TOMC-LAB2. Consumers perceived them similarly, only saltiness had a marked adverse effect [78]. 

Probiotics are generally carried through dairy products. However, the increased incidence of lactose intolerance, concerns over cholesterol, and the wide spread of new lifestyles (vegans and vegetarians) drove new researches toward non-dairy probiotic foods, such as fruits and vegetables, which are rich in vitamins, minerals, carbohydrates, fibers, and antioxidant compounds [126,127]. Recently, it has been shown that vegetable-derived products (i.e., fruits, fruits juices, cereals, and legumes) can act as carriers for positive microbes because of their intrinsic structure; thus, microorganisms can colonize pores, lesions, lenticels, and irregularities present on the surface [119]. Moreover, vegetables are also rich in prebiotic compounds, which protect probiotic microorganisms from the harsh GI tract conditions and are a source of nutrients that positively influences bacterial survival [128,129]. Actually, vegetable-based probiotic foods are available on the market. However, further studies are necessary to better understand the viability of selected strains in the human GI tract and their interactions with human microbiota. In vivo studies are required to assess if carried bacteria and the food matrix have a positive impact on human health. In this case, health claims could be proposed.

## 6. Conclusions

Table olives have a great impact on the economy of several countries. According to Bonatsou et al. [6], olives are considered in the food industry as the “food of the future”. Despite the many advances made, table olives are still produced according to ancient and local recipes, refusing the addition of starter cultures. Olive industries will face several challenges in the next future, including crop management, olive quality, production methods, and health issues. The application of starter cultures represents the main biotechnological challenge/innovation in this field. In this review the main criteria used for starter cultures selection are reported. LAB and yeasts are the main microbial groups studied and several strains have been characterized in order to develop new starter cultures. The use of autochthonous starter cultures is gaining attention since they offer several advantages in terms of adaptability to stressful niches and characterization of the final product, offering a link with the product origin. Another interesting aspect is the characterization of probiotic strains. This issue is the main research trend in this field since it responds to consumer demand for health-oriented products. The potential addition of probiotics in table olive fermentation on one hand give rise to new questions to be solved in terms of cost-effectiveness and acceptance by consumers, but on the other hand can improve the entire production process by positively affecting the aroma and sensory profile, product shelf-life, and by providing additional health-promoting properties to the consumers. Moreover, the development of probiotic table olives could have a positive economic impact, since this product is produced also in less developed countries. 

In our opinion, further studies are necessary to isolate and characterize more strains from different table olive cultivars in order to prepare autochthonous starter culture collections and produce healthy products with enhanced sensory characteristics. Additional researches are also needed to implement fermentation strategies to favor the survival and dominance of starter strains and develop new starters by combining LAB and yeasts, to mimic the natural microbiota of olives. Moreover, concerning probiotic strains, further validation in *in vivo* trials with more complex animal or human systems should be performed to gain a deeper understanding of their potential health-promoting features for humans. Finally, further studies should develop new approaches for the treatment of wastewater produced by table olive industries in order to have healthy eco-friendly products.

## Figures and Tables

**Table 1:** Olive processing methods according to the International Olive Oil Council (IOOC) [1].

Preparation Method	Process
Treated olives	It is applied to green olives, olives turning color, or black olives. Olive debittering is achieved through an alkaline treatment (lye 2.5%–3% w/v). Olives are then placed in brine (NaCl 10%–11% w/v) where the fermentation takes place and lasts 3–7 months. Fermentation is driven by lactic acid bacteria.
Natural olives	It is applied to green olives, olives turning color. or black olives. Olives are placed directly in brine. With a salt concentration of about 6%–10% (w/v). Oleuropein is removed through the enzymatic activities (mainly β-glucosidase and esterase) of indigenous microorganisms. The fermentation process can last 8–12 months and it is mainly driven by yeasts and lactic acid bacteria.
Dehydrated and/or shriveled olives	It is applied to green olives, olives turning color, or black olives. Olives are subjected or not to a mild alkaline treatment, preserved in brine, or partially dehydrated in dry salt and/or by heating.
Olives darkened by oxidation	It is applied to green olives or olives turning color. Olives are preserved in brine, fermented or not, and darkened by oxidation in an alkaline medium. They are stored in hermetically sealed containers and subjected to heat sterilization.
Specialties	Olives prepared in a different way than those above following traditional recipes.

**Table 2:** Characteristics and advantages in the use of selected starter cultures.

Properties	Characteristics	Advantages
**Safety**	Safe and stable activity Standardized activity Easy to manage and reproduce	Reproducibility Controlled and stable fermentation Continuous monitoring of fermentation
**Technological**	Ability to colonize olives surface (i.e., biofilm formation) Low demand for nutrients	Rapid and predominant growth High adaptation ability
	Growth at different pH (high/low) Salt tolerance Ability to survive/growth at low temperatures	Dominance during the fermentation
	Biodegradation of phenolic compounds Debittering activity (i.e., oleuropeinolytic activity) High acidification activity	Reduction of fermentation time Avoided use of chemicals (microbial biotransformation)
**Functional**	Antimicrobial activity vs. pathogens (i.e., bacteriocins production, competitive action on nutrients) Biocontrol agents vs. spoilage microorganisms (i.e., production of killer factors)	Protection from undesirable and/or pathogenic microorganisms Improvement of final product stability and shelf-life extension
	Enzymatic activities (i.e., lipase, alkaline/acid phosphatase, β-glucosidase) Vitamins production Production of aromatic compounds	Enhancement of organoleptic, nutritional and sensory profile of the final product
**Probiotic**	Survival under gastrointestinal conditions (i.e., low pH, gastric and pancreatic digestion, bile salts) Ability to adhere and persist in the intestinal mucosa Modulation of host immune system Antimicrobial activity against pathogens	Ensuring product safety Quality enhancement of the final product Production of a health-promoting functional food

**Table 3:** Main starter strains used for table olive fermentation.

Bacterial Starter Cultures	Cultivar	References
*L. plantarum*	Alorena	[40]
	Bella di Cerignola	[33,63,64,65]
	Carolea/Cassanese	[66]
	Conservolea	[41]
	Gordal	[40]
	Halkidiki	[67,68,69]
	Hojiblanca	[40,70]
	Kalamata/Chalkidikis	[62,71]
	Manzanilla	[40]
	Mele	[28]
	Nocellara del Belice/Nocellara Messinese	[66]
	Nocellara Etnea	[34]
	Picholine	[72]
	Pishomi	[42]
	Tonda di Cagliari	[39,61]
	Leccino	[44]
*L. pentosus*	Arbequina	[73]
	Conservolea	[41,52]
	Gordal	[55,74]
	Halkidiki	[67,68,69]
	Itrana	[15,35]
	Manzanilla	[40,75,76,77,78,79]
	Nocellara del Belice	[36,80]
	Nocellara Etnea	[34]
	Tonda di Cagliari	[39,61,81]
*L. paracasei*	Bella di Cerignola	[9]
*L. rhamnosus*	Giaraffa e Grossa di Spagna	[82]
**Yeast starter cultures**	**Cultivar**	**References**
*N. molendini-olei/C. matritensis/C. adriatica/C. diddensiae/W. anomalus/S. cerevisiae*	Taggiasca	[83]

**Table 4:** Main multi-starter strains used for table olive fermentation.

Multi-starter Cultures	Cultivar	References
*L. plantarum/L. pentosus*	Bella di Cerignola	[65]
	Halkidiki	[67,68]
	Nocellara Etnea	[34]
*L. plantarum/L. casei*	Nocellara Etnea	[84]
*L. plantarum/L. paracasei*	Giaraffa e Grossa di Spagna	[82]
*L. plantarum/L. paracasei*	Nocellara Etnea	[43]
	Nocellara Etnea	[34,85]
*L. plantarum/P. pentosaceus*	Green olives	[70]
*L. plantarum/E. faecieum*	Green olives	[70]
*L. paracasei/L. pentosus*	Nocellara Etnea	[34]
*L. pentosus/L. coryniformis*	Nocellara del Belice	[12]
*L. plantarum/L. paracasei/L. rhamnosus*	Giaraffa e Grossa di Spagna	[82]
*L. plantarum/L. paracasei/L. pentosus*	Nocellara Etnea	[34]
*L. plantarum/D. hansenii*	Conservolea	[53,86]
*L. plantarum/C. famata/C. guilliermondii*	Bella di Cerignola	[64]
*L. plantarum/S. cerevisiae*	Leccino	[53,86]
*L. plantarum/W. anomalus*	Cellina di Nardò	[53,86]
*L. plantarum/W. anomalus*	Bella di Cerignola	[33,65]
*L. plantarum/W. anomalus/L. pentosus*	Bella di Cerignola	[33]
*L. pentosus/P. membranifaciens*	Conservolea	[52,53]
*L. pentosus/C. boidinii*	Manzanilla	[87]
*L. mesenteroides/S. cerevisiae*	Kalamata	[53,86]
